# Hydroxylapatite and Related Minerals in Bone and Dental Tissues: Structural, Spectroscopic and Mechanical Properties from a Computational Perspective

**DOI:** 10.3390/biom11050728

**Published:** 2021-05-13

**Authors:** Gianfranco Ulian, Daniele Moro, Giovanni Valdrè

**Affiliations:** Centro di Ricerca Interdisciplinare di Biomineralogia, Cristallografia e Biomateriali, Dipartimento di Scienze Biologiche, Geologiche e Ambientali, Università di Bologna Alma Mater Studiorum, P. Porta San Donato 1, 40126 Bologna, Italy; daniele.moro@unibo.it

**Keywords:** bone/dental tissues, hydroxylapatite, biological apatite, collagen, quantum mechanics, density functional theory, molecular mechanics, crystal-chemistry, biological/inorganic interfaces, mechanical properties

## Abstract

Hard tissues (e.g., bone, enamel, dentin) in vertebrates perform various and different functions, from sustaining the body to haematopoiesis. Such complex and hierarchal tissue is actually a material composite whose static and dynamic properties are controlled by the subtle physical and chemical interplay between its components, collagen (main organic part) and hydroxylapatite-like mineral. The knowledge needed to fully understand the properties of bony and dental tissues and to develop specific applicative biomaterials (e.g., fillers, prosthetics, scaffolds, implants, etc.) resides mostly at the atomic scale. Among the different methods to obtains such detailed information, atomistic computer simulations (in silico) have proven to be both corroborative and predictive tools in this subject. The authors have intensively worked on quantum mechanical simulations of bioapatite and the present work reports a detailed review addressed to the crystal-chemical, physical, spectroscopic, mechanical, and surface properties of the mineral phase of bone and dental tissues. The reviewed studies were conducted at different length and time scales, trying to understand the features of hydroxylapatite and biological apatite models alone and/or in interaction with simplified collagen-like models. The reported review shows the capability of the computational approach in dealing with complex biological physicochemical systems, providing accurate results that increase the overall knowledge of hard tissue science.

## 1. Introduction

In vertebrates, bone is a dynamic connective tissue, which is responsible for various important functions, such as the protection of vital organs, structural support and motion, storage of calcium and phosphate, and haematopoiesis [1,2]. Generally speaking, a broad classification of the different bones in the human skeleton considers their morphology (long, short, flat, irregular and sesamoid), but other subdivisions exist, such as the distinction between woven and lamellar [2].

However, from the “material scientist’s” point of view, bone tissue is a heterogeneous composite material that, at its lowest hierarchal level, is made of an organic matrix (about 20–30 wt.%), an inorganic one (between 60 wt.% and 70 wt.%) and water (up to 10 wt.%). The organic part of bone is given mostly (90 wt.%) by collagen fibrils, of which type-I is the predominant one, formed by triple helix of two α_1_ and one α_2_ chains made of (Gly-X-Y)_n_ sequences (X and Y are commonly proline and hydroxyproline). Other collagens that are present in the organic matrix are type-V and type-XII.

**Figure 1:**
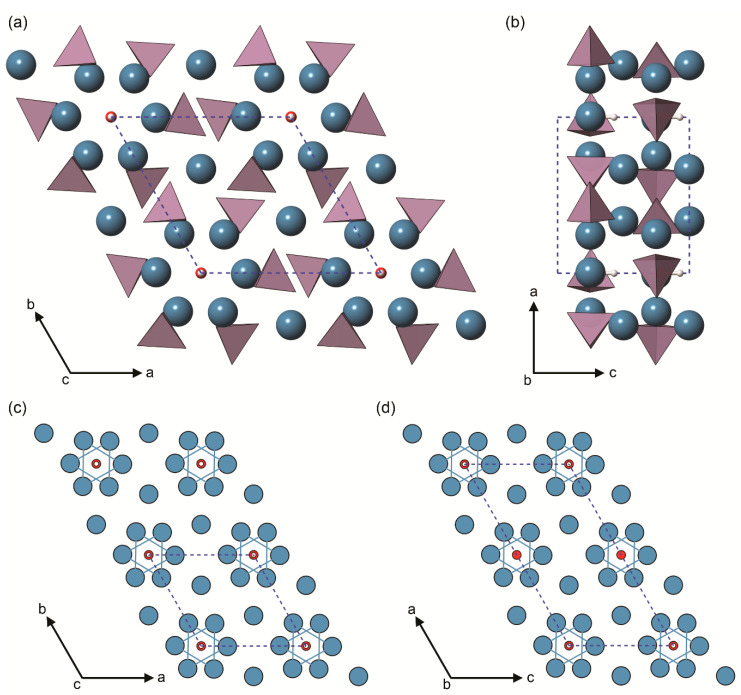
Structural models of hexagonal hydroxylapatite, OHAp, reporting phosphate groups PO_4_^3−^ as polyhedrons. (**a**,**b**) Different crystallographic views of the hexagonal polymorph (space group *P6_3_*) of the mineral unit cell. Panel (**c**) reports a simplified scheme of OHAp, highlighting the same alignment of hydroxyl groups in the channel of calcium ions in the hexagonal polymorphs. In panel (**d**), The unit cell was doubled along the *b* crystallographic axis to allow an easier comparison with the monoclinic polymorph of OHAp (space group *P2*_1_*/b*) reported whose OH alignment is alternated respect to panel (**c**) where the hydroxyl groups are all aligned in the same direction. In each panel, the blue dashed line represent the unit cell of the mineral. Colour coding for atoms: dark cyan–Ca; pink–PO_4_^3−^; red–O; white–H.

The inorganic part of bone and dental tissues is given by ‘biological apatite’, a mineral phase that is strongly correlated to hexagonal hydroxylapatite (labelled as OHAp, Ca_10_(PO_4_)_6_(OH)_2_, space group P6_3_/m, see Figure 1). Actually, the biological apatite in bone is a strongly “defective” OHAp mineral, because of a combination of different anionic/cationic substitutions and vacancies, which are generally expressed with the formula (Ca,X)_10_(PO_4_,Y)_6_(OH,Z)_2_. This inorganic matrix is mostly crystalline but may present a small fraction of amorphous phase [3].

This unique composition, together with the complex hierarchal structure of bone, plays a fundamental role in defining its peculiar static and dynamic mechanical properties, presenting at the same time both stiff (apatite) and tough (collagen) behaviours [4]. In fact, it seems that the apatite content in the connective tissue varies according to the function performed by the specific bone. For example, it is expected that agile animals (e.g., a cat) should have highly elastic bones, with more collagen than mineral, whereas the opposite occurs with big animals such as whales, where bones should have a much higher mineral content (ca. 80% by weight) [5].

Teeth are composed by three hard tissues, i.e., enamel, dentin and cementum, each exhibiting different composition, properties and functions. Enamel is the external layer of tooth, given by tightly packed (high density) crystals of hydroxylapatite (about 97 wt.%, low carbonate ion content), a small organic fraction (mostly amelogenin protein, 1.5 wt.%) and water (1.5 wt.%) [6,7]. The main function of the enamel is protecting the underlying dentin layer, which encapsulates the pulp, a highly vascularized and innervated soft tissue., Dentin, differently from enamel, is porous, composed by about 70 wt.% of carbonated hydroxylapatite, 20 wt.% of organic matrix and about 10 wt.% of water [6,8]. Cementum has a composition similar to that of dentin, and its function is attaching the tooth to the alveolar bone by anchoring the periodontal ligament. From experimental research, the elastic modulus of dentin and enamel are about 15 GPa and 80 GPa, respectively, which are stiffer than that of bone tissues, ranging between 0.3 GPa and 14 GPa [8]. Conversely, the tensile strength of bones (150 MPa) is much higher than that of both enamel (10 MPa) and dentin (100 MPa). A peculiar mechanical feature of the dentin–enamel joint, i.e., the interface between the two hard tissues, is that it is a stress-free region, as recently observed from measurements of residual stress on relaxed and compressed tooth samples [9].

There are several interesting reviews in literature focused on different aspects of bone tissues, biological apatite, and related topics [8,10,11,12,13,14,15,16,17]. The present work is intended to provide the reader with a different point of view, focusing on the crystalline, spectroscopic, mechanical, and surface properties of the biomineralized material. In fact, most of the properties of the different hard tissues (bone, enamel, dentine) in vertebrates are related to the subtle interplay between those of the mineral phase (biological apatite), those of the collagen matrix, and the apatite/collagen interface. Such knowledge can be obtained only by considering the nano- and sub-nanoscale components of inorganic and organic matrices of bone/hard tissues, in particular the atoms and molecules forming their structure. This atomic-scale data could be useful for:

- Obtaining new physical, chemical, and biochemical information of the complex features of the bone and dental tissues, which could aid understanding the hard tissues pathologies adverse effects (e.g., osteoporosis, osteosarcoma, etc.) and how to treat them.
- The development of new biomaterials with improved biocompatibility and bioactivity, aiming at obtaining a full restoration and rehabilitation of the damaged tissues.

Indeed, calcium phosphates, and in particular hydroxylapatite, are the most studied in the biomaterial field because of their chemical similarity with bones and teeth [8]. In this sense, it is worth distinguishing between “biological materials”, such as bone tissues, and “biomaterials”. In fact, according to the definition given by Williams almost a decade ago [18], “A biomaterial is a substance that has been engineered to take a form which, alone or as part of a complex system, is used to direct, by control of interactions with components of living systems, the course of any therapeutic or diagnostic procedure, in human or veterinary medicine”. Thus, while biological materials are those “simply” synthesised by the living organisms [19], a biomaterial needs to be accepted by the host, i.e., it must be biocompatible. In the materials science world, hydroxylapatite-based biomaterials are classified as bioceramics, a family comprising other polycrystalline materials, amorphous materials (glasses), and blends (glass/ceramics), that satisfy this biocompatibility requirement [17,20]. Nowadays, different OHAp-based formulations are commonly employed in various applications throughout the human body, for example bone-defect healing, fracture treatment, spinal surgery, dental fillings, and periodontal treatments [15,16,20].

The main subjects of this review are different computational approaches at the atomic scale to obtain information at the nanometre of hard tissues. These methods typically follow a bottom-up approach, which means that a system as complex as bone is decomposed in its simpler building blocks. After the different properties of biological apatite and collagen/organic fibrils are characterized, the further step is considering their interaction in the composite material, which needs further investigations. The results from atomic-scale simulations (from few to thousands of atoms) can then be used by other computational methods at both the mesoscale and the macroscale (engineering level), such as, for the sake of an example, finite elements modelling (FEM).

The objective of this work is providing the interested reader an innovative review based on a critical discussion of several computational investigations that are conducted with different degrees of physical-chemical accuracy on hydroxylapatite and more biological apatite models. Several works have been reported in literature on this subject, and here the focus is on the structural, vibrational, and mechanical properties of both stoichiometric and defective hydroxylapatite and the interaction between the mineral surfaces and the environment.

For the sake of logic path, clearness and completeness, before delving in the discussion of the results in literature, a brief summary of the main atomistic computational approaches, comprising some historical background, capabilities and limitations, is reported. To the authors’ knowledge, no specific review of computational studies at atomic scale can be found in literature devoted to the mineral phase of bone and dental tissues. To cite an example, almost a decade ago, Hamed and Jasiuk [21] provided an interesting summary of computational studies conducted with methods ranging from the micro- to the macroscale, such as FEM and micromechanics models. The cited authors reported just few examples of atomistic modelling, which were however focused on the organic fraction of bone tissues. Meanwhile, in the last ten years, more accurate models have been proposed, which are reviewed for the first time in the present work and could represent basic knowledge and a starting point for the interested readers.

## 2. Computational Methods

Computational science, born with the advent of electronic calculators around 1940 and grown since 1965 with the release of microprocessors [22], is a branch of science that uses numerical (mathematical) techniques to solve complex and/or large-scale interdisciplinary problems. In particular, computational methods aim at finding answers to physically, chemically and/or biological/medical relevant questions in several and manifold fields of research [23]. For this reason, and thanks to the exponential growth of the hardware computing power in the last twenty years, computational methods not only assumed a dominant role in modern science and engineering, but they have also carved out their own space in industrial applications.

Considering the bottom-up approach previously introduced, everything, from the simple gaseous molecules in the air to complex entities as a human body, is made of atoms. Starting from these simple building blocks, it is possible to model the behaviour of any system using mathematical methods in combination with the physical laws that rules the condensed matter.

From both the chemical, biological, and material science points of view, it is of utmost importance to provide an accurate physical description of the interaction between the atoms/molecules forming the system under investigation. It is worth understanding that the information gained from a simulation and the interpretation of its results can be only as reliable as the theory used to describe a system made of electrons and nuclei (quantum-mechanical problem). In other words, if prediction power is the target of the research, it is often mandatory starting from the sub-atomic scale of the electronic many-particle problem. However, for many questions of scientific or technological relevance, the phenomena of interest take place on much larger length and time scales. In the context of such challenge, the two main approaches that fulfil the accuracy requirements of the simulation are the quantum mechanical (QM) and molecular mechanical (MM) ones.

### 2.1. Ab Initio Quantum Mechanical Methods

The advent of quantum mechanics dates back to the first half of the 20th century, with its core being the Schrödinger equation, whose general expression is:

$$i\hbar \frac{\partial \Psi \left(r,t\right)}{\partial t}=\hat{H}\Psi \left(r,t\right)$$

where *i* denotes the imaginary unit, *ħ* the reduced Planck’s constant, Ψ(**r**, *t*) the wavefunction describing the electrons and nuclei of the system (whose variables are the spatial coordinates, **r**, and time, *t*) and *Ĥ* the Hamiltonian operator (sum of the kinetic energy and potential energy operators).

It is beyond the scope of the present work to provide a full description of the treatment of both the time-dependent and time-independent Schrödinger equation [24]. Here, it is just worth recalling that it can be solved analytically only for the hydrogen atom (single electron), hence numerical solutions based on the Hartree–Fock (HF) method [25,26] or density functional theory (DFT) [27,28] and other approximations (e.g., the Born–Oppenheimer approximation) are necessary to solve the many-body problem. The key ingredients of such approaches are:

- The way in which the electrons of the atoms are described, for example by using linear combinations of Gaussian-type orbitals (GTO), plane waves (PW) or numerical atomic orbitals (NAO).
- The choice of the Hamiltonian operator, which could be that formulated in the HF theory or a density functional.

This is why these methods are called ab initio or ‘first principle’: the only necessary knowledge is the type of atoms composing the molecular/crystalline system and a (reasonable) guess of their initial position coming from either a theoretical or experimental source.

The wave function obtained from HF theory accounts for up to about 99% of the energy of the system under consideration, if a sufficiently large basis set is employed. However, the ≈1% of energy not treated in HF, which is called correlation energy, is indeed very important for the proper description of physico-chemical processes. For instance, Hartree–Fock theory severely underestimates the bond dissociation energy due to the missing electron correlation [29,30]. To overcome this issue, several methods were developed to calculate this important term, which use the HF wave function as starting point, hence their name, post-HF methods. Among them, the configuration interaction (CI), many-body perturbation theory (MBPT) and coupled cluster (CC) are the most employed ones. Very briefly and with some approximation, these methods add an energy correction that typically assumes the form of an expansion series of terms of different orders (first, second, third, and so on). Thus, in principle, wave function theory can reach the “exact solution (i.e., physico-chemical accuracy)” by increasing the basis set size and the order of the post-HF methods. However, it must be stressed that post-HF corrections are very demanding in terms of computation power, hence they are typically limited to small systems (ca. 10 atoms) [29,30].

**Figure 2:**
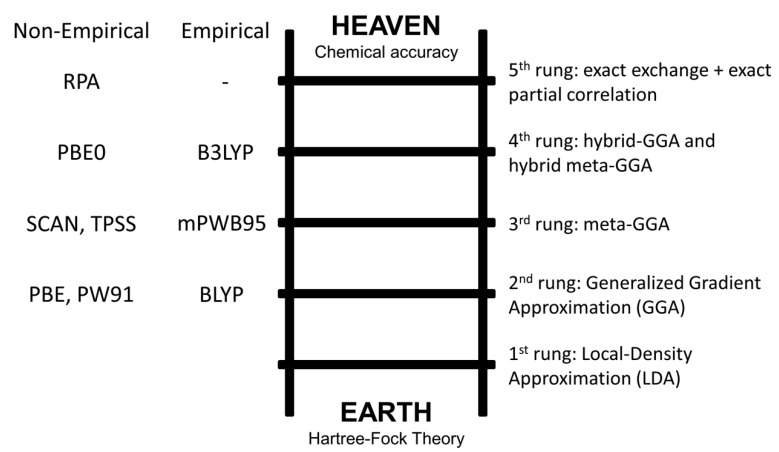
Jacob’s ladder of quantum mechanical methods in solid state and in quantum chemistry, representing the different level of accuracy of DFT methods [29].

The density functional theory, based on the Hohenberg–Kohn theorem stating that the ground state electronic energy of any system is determined completely by the electron density *ρ* [27], is one of the most diffuse approaches in both quantum chemistry and solid-state physics. The advantage of DFT over wave function methods resides in the fact that the complexity of the latter increases exponentially with the number of electrons, whereas the number of variables that describe the electron density are constant and does not depend on the size of the system. The only issue with DFT is that the functional that link the electron density to the ground state energy is not known. Hundreds of functionals have been developed, subdivided in non-empirical and semi-empirical ones [24,31,32]. However, in contrast to what is discussed for the wave function theory, it is not possible to improve the density functionals in a systematic way. A possible DFT hierarchy is given by the ‘Jacob’s Ladder’ depicted by Perdew (see Figure 2) [33]. Starting from the ‘Earth’ level, related to the HF theory, each rung contains new physical content that is missing in lower rungs, hence improved accuracy should be attainable at each higher level. A recent review of Mardirossian and Head-Gordon [24] elegantly summarized these topics, and the interested reader may find further details in the cited work.

Notwithstanding the employment of approximations, complex systems presenting hundreds of atoms (e.g., minerals with large unit cells, small proteins) could be treated in a reasonable amount of time only with adequate computing power and performances, in terms of both hardware (microprocessors, volatile and storage memory, network speed and so on) and related adapted software (numerical libraries, parallel coding, etc.). Using the words of Dovesi and co-workers [34], “it is a moving target; as the performance of the hardware improves rapidly, so should the software”. At present, some systems are still extremely difficult to treat at full ab initio level, thus it is important to know the limit separating what can be simulated with first principle methods and what requires more approximate methods (force fields, semi-empirical Hamiltonians, and so forth).

### 2.2. Classical Molecular Dynamics Methods

In atomistic simulations, the smallest unit in the system is the whole atom: this is the field of classical molecular dynamics (MD), sometimes referred to as molecular mechanics (MM). This approach uses empirical, meaning parametrised, representations of the interactions between particles, based on simple potential formulations (called force fields, FF) such as the following one where the energy of the system under investigation (*E*) is given by the sum of different terms, each one depending on several parameters [35]:

$$\begin{array}{c} E=\underset{bonds}{\sum }\frac{1}{2}k_{b}{\left(r-r_{0}\right)}^{2}+\underset{angles}{\sum }\frac{1}{2}k_{a}{\left(a-a_{0}\right)}^{2}+\underset{torsions}{\sum }\underset{n}{\sum }\frac{V_{n}}{2}\left(1+\cos\left(n\phi -\delta \right)\right)+\\ \underset{Lennard-Jones}{\sum }4\varepsilon_{ij}\left[{\left(\frac{\sigma_{ij}}{r_{ij}}\right)}^{12}-{\left(\frac{\sigma_{ij}}{r_{ij}}\right)}^{6}\right]+\underset{Coulomb}{\sum }\frac{q_{i}q_{j}}{r_{ij}} \end{array}$$

The first summation considers each bonded atom pair, with *k_b_* the stiffness of each bond, *r*_0_ the equilibrium bond length and *r* the bond length. The second summation runs over all the triplets of bonded atoms, with *a*, *a*_0_ and *k_a_* the bond angle, equilibrium angle and the stiffness of the angle, respectively. The torsional summation considers all dihedral angles described by quadruplets of bonded atoms and, because of the periodicity of the rotations about bonds, this term employs a cosine Fourier series. The dihedral angle *ϕ*, the phase of the angle δ and the stiffness of the dihedral *Vn* are the parameters describing this energy term. As it can be noted, the energy related to the system geometry (bond lengths, angles and torsions) is given by harmonic spring potentials. The last two summations are related to the non-bonded and electrostatic interactions, modelled as Lennard-Jones and Coulomb potentials, respectively. For the non-bonded interactions between atom *i* and atom *j*, with *i ≠ j*, the parameters *ε_ij_* and σ*_ij_* are the minimum interaction energy and the atomic distance for which the interaction energy is zero, respectively, whereas *r_ij_* is the interatomic distance. In the Coulomb electrostatic potential, *q_i_* and *q_j_* are the charges of the particles and *r_ij_* their distance. Hence, each addendum presents one or more parameters (*k_b_, r_0_, k_a_, a_0_*, *V_n_, δ*, *ε* and *σ*) that can be calculated and optimized with experimental, theoretical or both approaches. For example, the parameters of the AMBER [36] FF are obtained by a combination of both experiments (bond lengths, valence and torsional angles), ab initio simulations (torsional angles and atomic charges) and Monte Carlo liquid simulations (Lennard-Jones potential) [35]. Obviously, it is possible considering more complex formulations of the potential than the one previously reported, such as those of reactive force fields (e.g., ReaxFF [37]) and semi-empirical methods (such as density-functional tight binding, DFTB [38]), but the fundamental physics behind them are the same.

Typical applications for classical MD-based studies are within the realm of biomolecular simulations such as proteins, nucleic acids, transmembrane proteins dynamics [39], and even full viruses and bacteria [40,41,42]. However, even material science can benefit from the molecular mechanics approach, where specific FF were developed for instance for polymer chemistry, comprised of the same elements with similar functional groups as found for instance in peptides and proteins.

However, it should be pointed out that the capability of classical MD simulations in reproducing and predicting experimental results is controlled (i.e., limited) by both systematic and statistic errors. The formers are related to the inaccuracies of the underlying models, whereas the latter arise from the finite length of simulations.

## 3. Modelling Biological Apatite: From Nano to Macro-Scale

In the present chapter, some of the most recent data regarding the bulk properties of biological apatite (structural, vibrational and mechanical) will be presented, reviewed and discussed, showing also how these properties are strictly related from the biological, mineralogical and physico-chemical points of view.

### 3.1. Crystal Structure

Being an Earth crust mineral, hydroxylapatite (OHAp) is important in the geo-mineralogical field. OHAp is commonly found with a monoclinic lattice (space group *P*2_1_/*b*), which is thermodynamically more stable than the hexagonal polymorph. The monoclinic to hexagonal polymorphic transformation in stoichiometric OHAp was experimentally measured to occur at about 200 °C and 1 atm [43].

Probably because of the high computational costs, the first structural simulations of stoichiometric hydroxylapatite (OHAp) dates back only to 2006. In that year, three research papers were published, reporting ab initio investigations on the structure of hexagonal [44] and monoclinic [45] hydroxylapatite, and on the substitution of calcium with lead within the mineral lattice [46]. All these works were performed within the DFT framework with different computational approaches. For example, Zahn and Hochrein [45] employed the PW91 GGA functional, whereas Corno and co-workers [44] adopted the hybrid B3LYP Hamiltonian. All the resulting unit cell lattices were in very good agreement with respect to the experimental data, showing a maximum deviation of about 2% on the lattice parameters.

It is interesting, even fascinating, that the natural evolution of hard tissues “selected” the (highly ordered) hexagonal phase of hydroxylapatite as the inorganic component of bone tissues. As previously explained, at temperatures lower that 200 °C the monoclinic phase is thermodynamically preferred [43]. However, the hexagonal biological apatite is entropically stabilized by the presence of extensive atomic substitution and non-stoichiometry (e.g., vacancies) in Ca, P, and OH channel sites. Considering the latter sites, there are several known anionic substitutions of fluoride (in enamel, for example) and carbonate ions (ubiquitous in bony tissues) that can easily enter in the channel parallel to the **c**-axis and that lead to fluoro- and carbonated apatites, respectively, both as end-members and in mutual solid solutions [47,48]. In this perspective, with respect to stoichiometric OHAp, it is expected a deep influence of the complex crystal-chemistry (presence of inclusions/substituents/vacancies, even in traces) of biological apatite on the structural, vibrational and mechanical properties of both the mineral phase and the organic/inorganic composite. This observation can be obviously extended to the biomedical and biomaterials fields, where tailoring the biocompatibility and osteoconductivity properties of OHAp-like materials is of utmost relevance. One of the powerful features of theoretical simulations resides in providing more insights on the effects of compositional variations in the unit cell of OHAp crystal framework, one at a time (e.g., a single Mg^2+^/Ca^2+^ substitution) or in combination (e.g., one Mg^2+^/Ca^2+^ and one F^−^/OH^−^ substitutions).

However, modelling vacancies, substitutions and inclusion/interstitial defects within the hydroxylapatite crystal structure is not a trivial task in terms of both crystallography and needed computational resources. Regarding vacancies, they could bring to calcium-deficient hydroxylapatite, such as that present in the enamel [8], or they could occur at the O, OH or H sites. One of the first theoretical work that explored all these possible substitutions is the one of Matsunaga and Kuwabara [49], where the authors employed projector-augmented wave basis sets and the PBE functional to study the structural and electronic properties of 2 × 2 × 1 supercells of OHAp with Ca, O, OH, and/or H substitutions. According to the calculated formation energy of the defects, the authors indicated the hydroxyl group vacancies as the most favourable and that coupled H^+^ and OH^−^ substitutions could be possible above 1000 K, in agreement with experimental findings [50]. Bystrov and co-workers performed several studies on both substitutional and interstitial defects in hydroxylapatite. For example, they investigated by quantum mechanical means the different effects of O, H, and OH vacancies, the interstitial defects of hydrogen atoms and hydroxyl groups, the substitutions of Ca with either Sr, Mg, Mn, or Se and Si^4+^/P^5+^ substitutions [51]. A very interesting result was that OH vacancies could play a very important role in modifying the surface charge of hydroxylapatite, with possible effects in the interaction with living cells (e.g., osteoblasts). The same research group further investigated the effects of oxygen vacancies occurring at different sites to better understand the optical and photocatalytic properties of the mineral phase [52,53].

**Figure 3:**
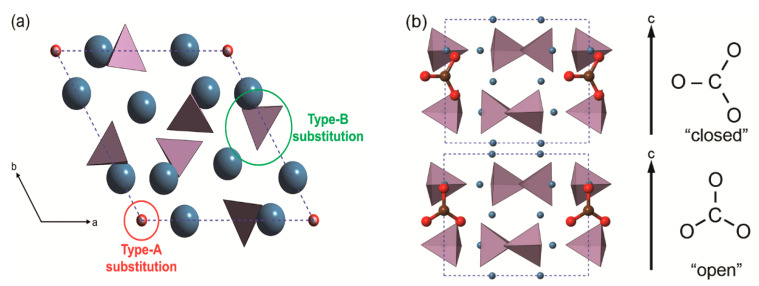
(**a**) Anionic sites interested in the carbonate ion substitutions in a single OHAp unit cell. It is worth noting that, due to *P6_3_* space group, the phosphate group are symmetrically equivalent. (**b**) Suggested configurations (“closed” and “open”) for the type-A carbonate ion. Colour coding for atoms: dark cyan–Ca; pink–PO_4_^3−^; red–O; white–H.

For what concerns inclusions/substitutions, those involving the carbonate ion are the most abundant. There are two anionic sites that the CO_3_^2−^ ion could occupy in the calcium apatite structure, one related to the hydroxyl group (type-A substitution) and the other to the phosphate ions (type-B substitution), as graphically shown in Figure 3. The CO_3_^2−^ ions substituting OH^−^ and/or PO_4_^3−^ lead to negatively or positively charged structure, respectively. In type-A carbonated hydroxylapatite models, the charge neutrality is commonly restored by removing an adjacent hydroxyl group, whereas type-B CO_3_^2−^ substitution allows several crystal-chemical solutions to obtain a neutral cell. Finally, both substitutions are simultaneously commonly found in the mineral phase of bone tissues [8], resulting in a mixed type-AB structure.

The first attempt to simulate the type-A carbonate apatite was that of Peeters and collaborators [54], who employed a simplified cluster model to simulate the lattice of a carbonated calcium apatite within the Hartree–Fock theory and with triple-ζ valence basis set. The model was not periodic and was limited to the hexagonal Ca^2+^ channel. The results suggested that the carbonate ion was oriented in the so-called “closed” (A1) configuration, consisting of one C–O bond perpendicular to the c-axis of the unit cell. In addition, it was shown that type A carbonate ion is almost free to rotate about the c-axis in the hexagonal calcium channel, a topic that would be reprised and rediscussed in the next years (vide infra).

Then, by using DFT and first principle pseudopotentials, Astala and Stott [55] investigated the effects of both type A and type B CO_3_^2−^ substitutions on the crystal structure, and different forms of charge compensation associated to the carbonate ion inclusions. In particular, for type-B carbonate ion substitutions, the authors considered three ways to compensate the extra negative charge: (i) removal of a single OH^−^ and a Ca^2+^; (ii) H^+^/Ca^2+^ substitution and (iii) creation of a calcium vacancy, together with a second CO_3_^2−^/ PO_4_^3−^ substitution. The study showed that B-type carbonate defects could be stabilized by the second proposed charge compensation, where it was also observed that the hydrogen atom bonds to a neighbouring phosphate, creating a HPO_4_^2−^ group.

Peeros and co-workers [56] performed molecular dynamics simulations using empirical interatomic potentials to study the type-A, type-B and mixed type-AB carbonate ion substitutions in OHAp. The authors employed the Nosé–Hoover thermostat in an NVT ensemble (a thermodynamic ensemble where the number of particles *N*, the simulation box volume *V,* and the temperature *T* are kept constant) with the thermostat set at 310 K, performing a simulation of 200 ps (time step of 0.2 fs). For type-A CO_3_^2−^ defect, the closed configuration was the most stable, with the carbonate ion free to rotate around the crystallographic c-axis. For the type-B substitution, the authors considered both sodium and potassium monovalent cations as substituents of a single calcium ion to restore the charge neutrality of the carbonated hydroxylapatite model. The results showed that the Na^+^/Ca^2+^ substitution is energetically preferred over the K^+^/Ca^2+^ one.

Rabone et al. [57] investigated the structure of hydroxylapatite and fluorapatite containing type-A or type-B defects by means of projector augmented wave (PAW) basis set [58] and the GGA functional PW91 [59], within the theoretical framework coded in the VASP code [60]. The structural and energy results at 0 K related to the orientation of the type-A carbonate ion in the unit cell closely follow those of Peroos et al. [56] obtained using molecular dynamics methods at 310 K. Different charge compensations for the type-B defects were considered. Interestingly, the results showed that for type-B fluorapatite the most stable structure is that presenting an additional fluoride ion near the carbonate substitution, whereas for hydroxylapatite a Na^+^/Ca^2+^ substitution is the one energetically preferred.

**Figure 4:**
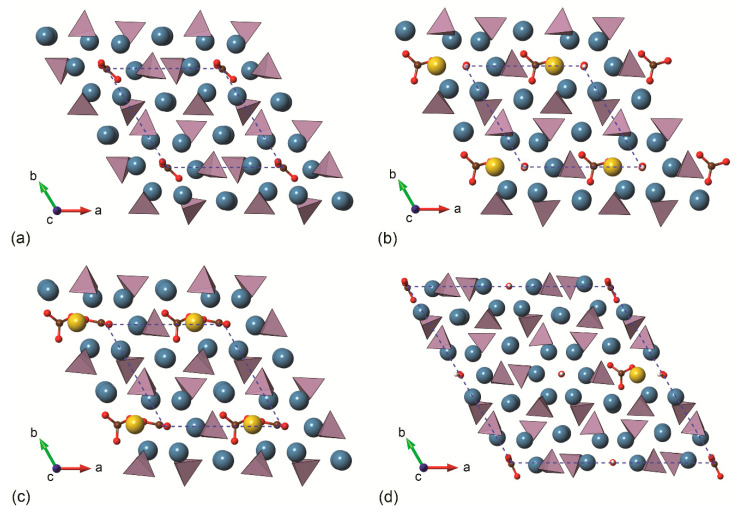
Views along the [001] crystallographic direction of different carbonated (hydroxyl)apatite models: (**a**) type-A CAp; (**b**) type-B COHAp with Na+/Ca2+ substitution; (**c**) type-AB CAp in a single unit cell; (**d**) type-AB COHAp in a 2 × 2 × 1 supercell. In each panel, the blue dashed lines represent the unit cell of the mineral. Colour coding for atoms: dark cyan–Ca; pink–PO_4_^3−^; yellow–Na; red–O; ochre–C; white–H.

More recently, Ulian and co-workers [61,62] reported DFT investigations on the type-A, type-B and type-AB carbonated (hydroxyl)apatite structures at the DFT/B3LYP level of theory and using all-electron double-ζ valence basis set. The positive charge of the type-B defect was neutralized by an additional Na^+^/Ca^2+^ substitution, considering ten models according to the cationic sites substituted by sodium. A graphical representation (ball-and-stick models) of the different crystal structures modelled by quantum mechanical simulations is reported in Figure 4. The simulations showed the same “close” configuration of the type-A CO_3_^2−^ ion observed in the previous studies, whereas the most stable type-B configuration was the one with the sodium ion near the oxygen vacancy created when the phosphate group was substituted by the carbonate ion, in agreement with experimental findings on synthetic samples [63,64]. For the first time, the complex type-AB carbonated hydroxylapatite structure was simulated at ab initio level, considering three 2 × 2 × 1 supercell models (with 175 atoms) according to the site occupied by the substituting CO_3_^2−^ ions [53]. The aims were (i) modelling a mineral phase that resembles the inorganic part of bone tissues from the crystal-chemical point of view and (ii) reaching a better understanding of the possible interactions between neighbouring carbonate groups. Albeit preliminary, the results on type-AB carbonated hydroxylapatite showed the distance between carbonate groups has different effects on the crystallographic axes of the mineral. In addition, it was observed that the structure is less stable if there is a small distance between the CO_3_^2−^ ions because of direct Coulomb repulsion, but also that the instability worsens if there is an excess separation between the different carbonate groups, in this case because of the lack of interaction. It was concluded that the most stable structure must lie between these two limiting distances considered.

Peccati and co-workers [65] specifically investigated at ab initio level the rotation of the type-A carbonate ion in both static (0 K) and dynamic (300 K) conditions. The focus of this work was on understanding the origin of the $P\bar{3}$ space group assignment to the type-A carbonated apatite. According to the symmetry operations of the space group, there is a disordering of the carbonate group around the **c**-axis, with 12 equivalent configurations that were characterized with a DFT/B3LYP-D* approach (the -D* indicated the inclusion of dispersive forces contribution to the total energy of the mineral). The results showed these configurations being almost equivalent from the energy point of view: the average cell parameters calculated according to Boltzmann’s well agree with the experimental data, confirming the assignment of $P\bar{3}$ space group. In addition, ab initio molecular dynamic simulations of Peccati and co-workers [65] confirmed the previous observations of Peroos et al. [56], i.e., the carbonate group can rotate almost barrierless around the c-axis.

It is worth noting that, despite the different computational approaches, all the cited studies agreed on the specific orientation of the type A carbonate ion within the unit cell. This topic was debated for a long time in literature, with some authors that experimentally observed the “close” configuration described above [63,66,67], while others reported the so-called “open” configuration where a C–O bond was parallel to the **c**-axis of the unit cell [58] (see Figure 3b). Both ab initio static (at 0 K) and molecular dynamics (at 300 K) simulations clearly demonstrated that the preferential type-A carbonate ion configuration is the “close” one [54,55,57,61]. Further investigations showed the ability the CO_3_^2−^ ion to freely rotate about the c-axis, but it was suggested that the energy barrier for the rotation of the carbonate ion plane about the a-axis may be too high to be easily overcome at room temperature, thus hindering the possibility to observe the “open” configuration in standard conditions of pressure and temperature [56,65].

Some of the most recent structural results (lattice parameters and bond lengths) of hydroxylapatite, type-A carbonated apatite and type-AB carbonated hydroxylapatite are reported in Table 1, whereas a comparison with the mineral phase of bone tissues (bone, enamel and dentin) is presented in Table 2. For OHAp, the variations of the unit cell parameters and bond lengths obtained with theoretical methods are within 0.42% and 1.0%, respectively, in comparison with with both synthetic and natural samples. Somewhat larger deviations (between 2% and 3%) were calculated considering carbonated (hydroxyl)apatite models, but they could be due to the high substituent content with respect to the experimental samples. For example, taking enamel as the reference for comparison (Table 2), in the single cell of type-B COHAp the carbonate and sodium contents are very high, and the lattice parameters deviated for up to 0.45%. Conversely, when the type-AB COHAp supercell model (2 × 2 × 1) was employed, the results were more in line with the experimental ones. Hence, to proper model complex crystal structures as biological apatite, it is strongly advisable to adopt large unit cell models to accommodate different anionic/cationic substitutions in a concentration closer to the experimental data. This is not a trivial task, because not only the size of the model increases, as does the computational power required, but also the number of possible crystallographic sites where the substitutions may occur.

### 3.2. Vibrational Properties

Infrared (IR) and Raman spectroscopies are two key analytical techniques that are widely used to characterise both the mineral and the organic components of bone and dental tissues [74,75,76,77,78,79,80,81,82], and also synthetic biomaterials employed in medicine for their regeneration/replacement [3,9,66]. Much information on the nature of hard tissues can be extracted from IR/Raman spectra of biological specimens, such as the degree of crystallinity, the Ca/P molar ratio, the presence and the kind of carbonate ions in the mineral phase [83,84], the type of cross-links between collagen fibrils [76,79], and many others that will be described in the following.

Both techniques investigate the vibrational motion of atoms within biomolecules (e.g., from simple amino acids to complex proteins) and inorganic solids (crystalline and amorphous). Each vibrational mode, which could be bond stretching, in and out-of-plane angle bending, wagging and twisting (change in angle between a bond and a plane, and between two planes, respectively), may give rise to a band (or peak) in the spectra when excited. The physics behind the generation of the bands visible in the vibrational spectra is different in IR and Raman spectroscopies. Very briefly, in the first method, when the sample is irradiated by infrared light in IR spectroscopy, this radiation will be absorbed if the specific (vibrational) motion with specific frequency is accompanied by a change of the dipole moment of the system. Instead, Raman spectroscopy is related to the inelastic scattering of monochromatic light, a phenomenon with very low probability of occurrence (about one event every 10^6^ photons). The energy difference between the inelastically scattered photons and the elastically scattered ones is related to the vibrational frequency of irradiated biomolecules and inorganic solids by the Planck–Einstein relation, i.e., *E* = *hν = hc/λ*, where *E* denotes the photon energy, *ν* the frequency, and λ the wavelength. In either spectroscopy, the position of the bands in the spectra is characteristic of the overall configuration of the atoms (chemical environment) and representative of specific chemical functional groups.

From the historical point of view, the ab initio calculation of IR and Raman spectra for solid system dates back to the first half of the Nineties [85], mostly confined within the Density Functional Perturbation Theory and using only plane wave basis sets. In the same period, the Fourier transformation of the velocity autocorrelation function obtained from the trajectory of molecular dynamics simulations was also a widely adopted method of computing vibrational spectra. In the first years of the 2000s, other algorithms were proposed and implemented in several first principle codes [86,87]. Although this kind of computational investigation is relatively young, especially at the ab initio level because of the high computational resources needed, it already provides very accurate results in the analysis of several inorganic systems. Indeed, this is an inestimable tool that could help experimentalists in assigning specific bands in complex spectra as those of bone and dental tissues. In the following, only some of the most relevant theoretical studies related to hydroxylapatite and biological apatite will be presented.

In crystalline solids, the number of vibrational modes is 3*N*–3, with *N* the number of atoms in the unit cell [88]. For hydroxylapatite (44 atoms in the unit cell), there are 129 vibrational degrees of freedom, whose complete characterization was initially performed by Corno and co-workers [44] and later by Ulian et al. [89]. In both cited works, the same theoretical approach was employed, namely the hybrid functional B3LYP and GTO basis sets for all atoms but calcium, which was a small-core pseudopotential in the work of Corno et al. [44] and an all-electron basis set in the study of Ulian and co-workers [89]. More recently, a small revision of this analysis was provided, including the contribution of dispersive forces in the quantum mechanical treatment [90]. According to the performed analyses, the vibrational modes Γ_vib_ of stoichiometric OHAp are subdivided in the following irreducible representations (IRREPs):

Γ_vib_ = 21*A* + 22*B* + 21*E*_1_ + 22*E*_2_,

of which 63 modes (21*A* + 21*E*_1_) and 107 modes (21*A* + 21*E*_1_ + 22*E*_2_) are active in IR and Raman, respectively, whereas *B* modes do not give any signal in both spectroscopies. For the sake of clearness, *E*_1_ and *E*_2_ modes are doubly degenerate, which means each of these modes are related to two vibrational motions of atoms having the same vibrational frequency. However, the typical IR/Raman analysis of the mineral phase of bone tissues focuses on the phosphate ion modes, which are subdivided as symmetric O–P–O bending (labelled as ν_2_, 2*A* + 2*E*_1_ + 2*E*_2_), asymmetric O–P–O bending (ν_4_, 3*A* + 3*E*_1_ + 3*E*_2_), symmetric P–O stretching (ν_1_, 3*A* + *E*_1_ + *E*_2_) and asymmetric P–O stretching (ν_3_, 3*A* + 3*E*_1_ + 3*E*_2_).

**Figure 5:**
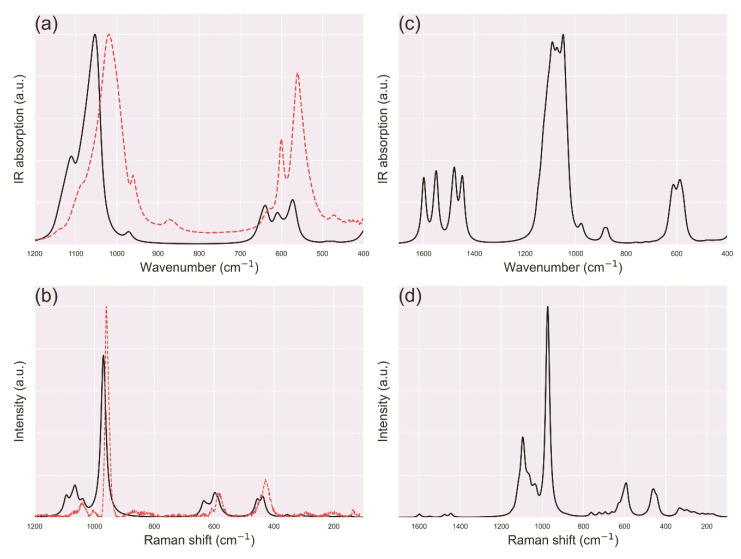
Simulated IR and Raman spectra of (**a**,**b**) hydroxylapatite and (**c**,**d**) type-AB carbonated apatite as obtained from DFT/B3LYP-D* simulations [72,90], plotted as black lines. The red dashed lines are experimental IR and Raman measurements reported in literature [74,92,93].

A summary of the vibrational analysis on stoichiometric hydroxylapatite OHAp is reported in Table 3, together with the irreducible representation (IRREP) of the different modes. It can be noted that the simulation results are in very good agreement with the experimental analyses carried out with IR and Raman spectroscopies of single-crystal synthetic and human dentin and enamel bone samples [74,91,92]. The simulations were performed on a perfect single-crystal of OHAp, whereas defects and impurities are typically present and observed in both synthetic and natural samples, which explains the small variations between the calculated vibrational frequencies and those experimentally obtained. A graphical comparison between the IR and Raman spectra of OHAp is shown in Figure 5, as simulated by Ulian and Valdrè [90] at the DFT/B3LYP-D* level of theory. It can be noted that, compared with the experimental data [74,92], the quantum mechanical results are slightly blue shifted, meaning the calculated vibrational frequencies are slightly higher. However, the intensities and the overall shape of the signals are well described by the adopted simulation approach.

**Figure 6:**
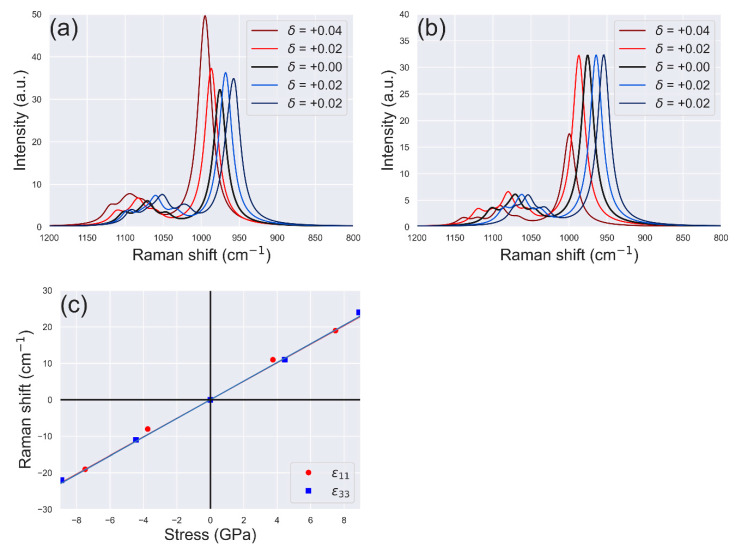
Simulated Raman spectra of hydroxylapatite, between 800 cm^−1^ and 1200 cm^−1^ (ν_1_ phosphate band), under the effect of uniaxial deformation (**a**) *ε*_11_ and (**b**) *ε*_33_, with *δ* representing the extent of deformation (adimensional number). (**c**) Dependence of the position (Raman shift) of the ν_1_ phosphate band on the applied uniaxial stress.

Theoretical analyses were also conducted on carbonated (hydroxyl)apatite models, where the carbonate ion presents two in-plane O–C–O bending modes (ν_4_), an out-of-plane bending mode (ν_2_), a symmetric stretching mode (ν_1_) and two asymmetric stretching modes (ν_3_). As previously introduced, the ${\mathrm{CO}}_{3}^{2-}$ vibrational signals fall at different wavenumbers according to the site (A or B) occupied by the anion within the apatite framework, because of the different chemico-structural environment surrounding the substituting ion. In this sense, the quantum mechanical simulations are an invaluable tool to carry out this kind of investigations, as it is possible to both decouple and couple the effects of type-A and type-B substitutions, aiming at a better comprehension on how the interaction between the ${\mathrm{CO}}_{3}^{2-}$ ions affect the overall vibrational spectrum. For example, Ulian and co-workers [71,72,89] performed this kind of analysis firstly considering the type-A and type-B defects separately, then they simulated the co-presence of the two ${\mathrm{CO}}_{3}^{2-}$ defects in different C(OH)Ap models, with or without hydroxyl groups (vide supra). The computational approach was the same adopted for the investigation of stoichiometric OHAp to obtain a comprehensive picture on how the carbonate ions modify the vibrational spectrum of the mineral. An example of the IR and Raman spectra calculated for type-AB carbonated apatite is reported in Figure 6c,d.

At the same time, Yi and co-workers [94,95] conducted similar simulations of several apatite models containing either type-A or type-B carbonate defects, but adopting a different theoretical framework, i.e., PWscf and PHonon codes [96], GGA functional and plane waves with the ionic cores described by ultra-soft pseudopotentials. In particular, the crystal-chemistry of the selected models was different in the mechanism to restore the charge neutrality required by the type-B carbonate substitution. In fact, while Ulian and co-workers [62] employed a Na^+^/Ca^2+^ substitution in line with the typical biological apatite [8], the approach followed by Yi and co-workers [94,95] was more geologically oriented, with inclusion of a monovalent anion (OH^−^ or F^−^) near the type-B carbonate ion. The reported vibrational frequencies for the type-A and type-B ${\mathrm{CO}}_{3}^{2-}$ ion are in line with the theoretical ones of Ulian and co-workers [62,89], considering the different crystal-chemistry of the apatite models, and the experimental findings of Suetsugu et al. [97].

The theoretical results related the carbonate ion vibrational modes are reported in Table 4, and compared to experimental analyses carried out using different techniques (FTIR, μ-FTIR and Raman) [98,99,100]. It can be noted that the simulated normal modes are all close to the experimental evidence, with some variation due to the different quantum mechanical approach used. For example, the combination of hybrid B3LYP functional and Gaussian-type orbitals basis sets resulted in a systematic blue shift with respect to the data collected by IR/Raman methods [57,84]. Conversely, a red shift (lower frequency) was observed in the case of GGA functional and plane wave basis sets [89,90]. As shown in recent literature [101], this kind of differences in both DFT functionals and atomic basis sets has an important influence on the calculation of structural and vibrational properties. However, the frequency difference between the asymmetric C–O stretching modes (Δν_3_ = ν_3b_ − ν_3a_), which is one of the parameters employed to discriminate between type-A and type-B carbonate ions, is well described by the simulations. For example, Δν_3_ is about 90 cm^−1^ for type-A ${\mathrm{CO}}_{3}^{2-}$ [62,94], in excellent agreement with the experimental value of 93 cm^−1^ [98]. Type-B carbonate ion substitutions are more prone to variations in terms of IR/Raman spectroscopy because of the vibrational modes are sensitive to the charge compensation; in other words, different crystal-chemical balancing methods lead to significant blue or red shift of the signals. For the sake of a comparison, the Na^+^/Ca^2+^ substitution provides Δν_3_ = 81 cm^−1^ [62], whereas further inclusion of fluoride of hydroxyl ions results in Δν_3_ of about 40 cm^−1^ [94].

### 3.3. Mechanical Properties

It is well-known that biological apatite and (tropo)collagen form a complex composite in bone, dentin, etc., presenting remarkable mechanical properties [102]. For instance, the elastic behaviour of bone tissues is strictly related to those of the mineral and organic components alone and to the structure–property–function relationships between them. It is worth recalling, however, that bone and dental tissues may contain up to about 10–15 wt% of water [8], which is expected having an influence on the overall elastic behaviour of this biological composite. In addition, it was recently suggested that, in the context of bone regeneration mechanisms, the activity of osteoblasts is triggered by biological/biochemical signals emitted by the transduction of mechanical ones [103]. Macroscopically, it was shown that the Young’s modulus (*E*) of cortical bone varies from 8 to 24 GPa, with hydroxylapatite presenting *E* ≈ 130 GPa and collagen a (tangent) modulus of ca. 1.25 GPa [5]. This extremely broad range of mechanical behaviour has to be characterized by considering the mineral and organic components alone and their structure–property–function relationships (interface) at nanometre and atomic levels. This kind of knowledge is of utmost importance not only to evaluate the elastic properties of hard tissues and to develop and create new biomaterials, but also to understand their diseased states, such as those induced by osteoporosis and other bone pathologies [104].

Considering the mineral phase of bony tissues, several mechanical properties could be described by the elastic moduli (or constants) matrix, **C**. The elastic constants measure the proportionality between strain and stress in a crystalline material within the Hook’s law limit, which means that the strain must not be too large. The result of the elastic analysis is a fourth-rank tensor that is simplified in a 6 × 6 matrix (2nd-rank tensor) according to the notation proposed by Voigt [105]. The expression relating the stress *σ* and the strain *ε* in a crystalline material, σ = Cε, is then, by using the matrix notation, of the form:

$$\left[\begin{array}{c} \sigma_{11}\\ \sigma_{22}\\ \sigma_{33}\\ \sigma_{44}\\ \sigma_{55}\\ \sigma_{66} \end{array}\right]=\left[\begin{array}{cccccc} C_{11} & C_{12} & C_{13} & C_{14} & C_{15} & C_{16}\\ C_{21} & C_{22} & C_{23} & C_{24} & C_{25} & C_{26}\\ C_{31} & C_{32} & C_{33} & C_{34} & C_{35} & C_{36}\\ C_{41} & C_{42} & C_{43} & C_{44} & C_{45} & C_{46}\\ C_{51} & C_{52} & C_{53} & C_{54} & C_{55} & C_{56}\\ C_{61} & C_{62} & C_{63} & C_{64} & C_{65} & C_{66} \end{array}\right]\left[\begin{array}{c} \varepsilon_{11}\\ \varepsilon_{22}\\ \varepsilon_{33}\\ \varepsilon_{44}\\ \varepsilon_{55}\\ \varepsilon_{66} \end{array}\right]$$

where *C*_ij_ are the elements of the elastic constant’s matrix C. Since C is symmetric, *C*_ij_ = *C*_ji_, thus there are 21 independent elastic constants. In general, the elastic constants for a three-dimensional system are defined as the second derivative of the Gibbs free energy, *G*, with respect to the strain ε, according to the following expression:

$$C_{ij}=\frac{1}{V}\frac{\partial^{2}G}{\partial \varepsilon_{i}\partial \varepsilon_{j}}$$

However, from the computational point of view they are commonly calculated considering the lattice energy (*U*) in place of *G*, and the effects of both pressure and temperature are included in the treatment *a posteriori* [106]. If the crystalline system under analysis is symmetric, the number of independent elastic moduli could be reduced. For example, hexagonal hydroxylapatite has only five independent terms:

$$C=\left[\begin{array}{cccccc} C_{11} & C_{12} & C_{13} & 0 & 0 & 0\\ C_{12} & C_{22} & C_{13} & 0 & 0 & 0\\ C_{13} & C_{13} & C_{33} & 0 & 0 & 0\\ 0 & 0 & 0 & C_{44} & 0 & 0\\ 0 & 0 & 0 & 0 & C_{55} & 0\\ 0 & 0 & 0 & 0 & 0 & C_{66} \end{array}\right]$$

where *C*_66_ = (*C*_11_ − *C*_12_)/2.

Recently, Ulian and Valdrè [68] made a comparison between the elastic moduli of hydroxylapatite obtained by different approaches within the DFT framework (at 0 K). An excerpt of the results is here presented in Table 5, together with previous theoretical and experimental analyses [107,108,109,110]. The reported analysis focused on two important technical details for both solid state physics and molecular simulations: (i) the choice of the basis set for the description of the atoms in the structure and (ii) the inclusion of dispersive forces. Two basis sets were selected, a linear combination of plane waves (PW) and a linear combination of local atomic orbitals, represented as Gaussian-type orbitals (GTO). It is not within the scope of the present paper to deal with the advantages and disadvantages of these methods, which can be found by the interested reader in dedicated literature [101,111]. The results showed that the elastic moduli calculated with plane wave PW basis sets are generally in better agreement with the experimental data than those obtained with GTO basis sets. The C matrix elements calculated with the latter approach are higher than the experimental ones, meaning an overestimation of the mechanical properties. Very briefly, this difference resides in the Hellman–Feynman theorem and in the local nature of Gaussian-type orbitals (i.e., the basis sets are dependent on the nuclei position), which would require the evaluation of the Pulay forces (the derivative of the basis set with respect to the atomic position). This step is generally computationally very intensive, and some quantum mechanical codes do not provide it yet.

Pezzotti [112] proposed an interesting experimental method that exploits Raman spectroscopy, cathodoluminescence and mechanical properties to characterize the residual stress stored in minerals and materials [9,112,113,114], including composites as complex as bone and dental tissues [3]. For example, at the experimental level, it was found that the interface between dentin and enamel in teeth is characterized by a zero interfacial stress [112]. This combined approach can discern the variations of both the collagen and mineral components of bone tissues, because the most intense Raman peaks of each phase is well separated in the spectra, and the vibrational spectroscopy is extremely sensitive to variations in the local chemical environment near the vibrating group. This novel stress analysis is able to map the piezo-spectroscopic ultra-structures of biomaterials and tissues. However, to be effective, the method needs atomic-scale information of the material, in particular how the Raman spectra change when the sample is under the effect of external stress. Hence, the approach has to be properly calibrated, either by experimental or theoretical means.

In this context, a theoretical investigation on the effect of mechanical stress on the Raman bands of hydroxylapatite was recently reported [84]. In the cited work, the authors simulated different strained configurations of the mineral, according to uniaxial and biaxial stress and calculated the Raman spectra for each of them. With the knowledge of the second-order elastic constants of the mineral, it was possible to calculate the piezo-spectroscopic components of selected Raman bands, which were in very good agreement with the previous experimental calibration of Pezzotti [114]. For the sake of an example, Figure 6 reports the simulated Raman spectra in the range 800–1200 cm^−1^ related to the following *ε*_11_ and *ε*_33_ elastic deformations along the x-axis and z-axis, respectively:

$$\varepsilon_{11}=\delta \left(\begin{array}{ccc} 1 & 0 & 0\\ 0 & 0 & 0\\ 0 & 0 & 0 \end{array}\right)\;\varepsilon_{33}=\delta \left(\begin{array}{ccc} 0 & 0 & 0\\ 0 & 0 & 0\\ 0 & 0 & 1 \end{array}\right)$$

with *δ* being an adimensional factor between ±0.04. It can be noted that the most intense band of hydroxylapatite (*ν*_1_ PO_4_) is shifted when the mineral is under uniaxially deformation, with respect to the equilibrium configuration (unstressed, *δ* = 0.00). Other bands are also affected, but it is commonly used the selected one because it is the most intense signal in the spectra. The same method was applied to IR spectroscopy as well [90], using in that case the *ν*_3_ phosphate band. The provided data could be useful in future to calibrate and extend the stress analysis method to IR spectroscopy also.

Thus, the second-order elastic moduli provide the directional mechanical behaviour, showing the possible anisotropy of the system under investigation. Other non-directional elastic properties can be obtained considering the equation of state (EoS) of the material, which correlates the unit cell volume *V* with applied hydrostatic pressure *P*. There are different PV-EoS formulations, such as the Murnaghan [116] and the Birch–Murnaghan [117], with the latter being:

$$P=\frac{3}{2}K_{0}\left(\eta^{7}-\eta^{5}\right)\left[1-\frac{3}{4}\left(4-K^{\prime }\right)\left(\eta^{2}-1\right)\right]+P_{0}$$

with

$$\eta ={\left(\frac{V}{V_{0}}\right)}^{-1/3}$$

where *K*_0_, *K*′, and *V*_0_ are the bulk modulus, the pressure first derivative of the bulk modulus and the unit cell volume, respectively, at 0 GPa. These parameters are experimentally obtained by fitting the EoS with unit cell data obtained at different pressure states.

This least square problem can also be solved theoretically from the computational point of view, using the same experimental equation of state formulations or volume-integrated ones (see for instance [118]). Several static (0 K) quantum mechanical studies were conducted to evaluate the equation of state of hexagonal hydroxylapatite, finding bulk moduli in the range 86 GPa–115 GPa, according to the selected computational parameters such as functional, basis sets and other settings [119,120,121]. Recently, this approach was extended to type-A carbonated apatite [71,122] and type-AB carbonated apatite [72], to understand how the different ${\mathrm{CO}}_{3}^{2-}$ ions affect the mechanical properties of the mineral. These studies reported that, with respect to the bulk modulus of hydroxylapatite (*K*_0_ = 115 GPa), the type-A and type-AB carbonate ion substitutions lower this value to 108 GPa and 104 GPa, respectively, in good agreement with the very few available experimental data [123].

It is worth noting that a proper comparison with the experimental findings should include the thermal effects, especially when dealing with structural and elastic properties of minerals, inorganic and organic materials. If the system has a small number of atoms (2–100) and/or some symmetry, it is possible to combine the *PV* knowledge from the equation of state with the vibrational properties of the material at different unit cell volumes in the so-called quasi-harmonic approximation. Very briefly, this is a solid-state approach that uses statistical mechanics formulations (harmonic approximation) to obtain several thermodynamic properties of a system. However, the harmonic approximation fails in describing features such as thermal expansion because, within this method, the atomic positions are independent on temperature. The quasi-harmonic approximation solves this issue by including an explicit dependency of the phonon (vibrational) modes on the unit cell volume of a solid phase. Full details of this approach are beyond the scope of the present review, but the interested reader could find the basic theory, formulations and different kind of implementations in dedicated works [124,125,126,127,128,129,130,131]. When applied to hydroxylapatite and carbonated apatite models [71,72,121], the quasi-harmonic approximation provided results in even better agreement with the experimental data than the data calculated at 0 K [123]. Another solution is employing thermodynamic ensembles able to control the temperature and the pressure of the mineral phase [132,133,134], both in ab initio molecular dynamics (AIMD) and in classical molecular mechanics.

At present, the elastic properties of both collagen and protein-biological apatite interface are beyond the capabilities of ab initio, first principle simulations because of the huge amount of atoms involved. On the contrary, this is the spatial and temporal domain that can be successfully tackled by means of molecular mechanics approaches, or even finite element modelling [135]. At the atomistic level, about a decade ago, Dubey and Tomar performed several simulations of collagen/(hydroxyl)apatite models to investigate the mechanical properties of the composite at atomistic level [136,137,138,139,140,141,142,143]. To cite an example, the authors investigated how the tensile and shear loads affect the collagen fibrils, modelled as a staggered arrangement of both tropocollagen (PDB ID ‘1YGV’ [144]) and hydroxyl platelets [140]. Different supercell models, with sizes from 1 nm to 20 nm in length, were subjected to uniaxial deformations in two chemical environments (vacuum and water) [145]. To simulate the inorganic/organic framework, a combination of the well-known CHARMM force field [146] and an interatomic potential specifically developed for hydroxylapatite [147] were adopted, and the elastic behaviour of the different composites were analysed to uniaxial strain up to 20%. It was found that the failure mechanism was mainly driven by shear stress, with the failure occurring in a ductile fashion. In addition, and very importantly, the analyses revealed that water molecules act differently as a lubricant or as a glue between adjacent tropocollagen molecules when the loading is of tensile or shear type, respectively.

Nair and co-workers developed a full-atomistic model to perform a systematic study of the mechanical behaviour of bone from a fundamental and molecular point of view [148]. In the cited work, conducted at the molecular mechanics level with the LAMMPS code [149], the authors modelled the collagen protein using the 3HR2 conformation reported on the Protein Data Bank [144] and the CHARMM force field parametrization [146]. The latter was extended to include the necessary parameters to simulate hydroxylapatite/collagen composite. For OHAp, the employed bond, angle and dihedral parameters were those calculated by Hauptmann and collaborators [147] from both quantum mechanical and empirical results. Different geometries of the tissue at the atomic scale were adopted, considering several degrees of mineralization (mineral density, intended as inorganic/organic ratio). The proposed model correctly predicted the mechanical properties of mineralized fibrils at the macroscopic scale, and provided fundamental insights on the possible mechanisms of deformation at the nano-scale and on the load transfer between the organic (collagen) and inorganic (mineral) components of the bone composite. For example, it was suggested that hydrogen bonds and other non-covalent interactions (e.g., salt bridges) play an important role in the load transfer mechanism between collagen and the mineral. In addition, the results of the molecular mechanics modelling suggested the OHAp crystals have an extremely small thickness of about ~15 × 3 × 1.6 nm^3^, in agreement with the typical elongated shape found in experimental bone and dental tissues [8].

More recently, Fielder and Nair [150] investigated with the same molecular mechanics framework the effect of water and mineral content on the mechanical properties of apatite/collagen/water system through uniaxial tensile deformation along the fibril length, considering three models with different mineral density (0 wt.%, 20 wt.% and 40 wt.%) and water content (up to 4 wt.%). The authors focused on the stress versus strain behaviour and Young’s modulus (*E*) of the composite, including a comparison between fibril gap/overlap regions formed during deformation. The results showed that the *E* value of the 40 wt% mineralized fibrils is almost double the difference in the tensile Young’s modulus of the non-mineralized ones, and this behaviour seems independent of the water content in the composite. However, the water content may have a deep influence on the deformation properties of bone-like composite model. In fact, according to the degree of hydration, the mineralized gap regions may be stiffer (H_2_O content ca. 2 wt.%) or more easily deformed (water content of about 4 wt.%).

In general, it is not straightforward comparing in a direct way the elastic properties obtained from quantum mechanics and classical mechanics. This is due to the different scales of the investigated models, ca. 1–2 nm at QM level and about 10–100 nm with molecular mechanics modelling, the latter often including both the organic and inorganic components of bone and dental tissues. For this reason, the comparison can be carried out only on the mineral phase of hard tissues. In this perspective, a good agreement was found between the bulk modulus of stoichiometric hydroxylapatite calculated from ab initio quantum mechanical data at 300 K (109 GPa) [121] and from force field calculations at the same temperature (100 GPa) [148]. This result suggests that the parametrization of the OHAp force field is adequate for the simulations of large-scale models of this mineral phase. Unfortunately, at the moment, the absence of mechanical simulations of the organic/inorganic composite performed at QM level hinders the same assessment, which could be of great importance because, in molecular mechanics simulations, different combinations of force fields for OHAp and protein are employed.

## 4. Biological Apatite and Interaction with the Bioenvironment

So far, the focus of the present review was on the bulk properties of biological apatite that can be studied by means of various and different theoretical approaches. However, as subtly anticipated in the previous chapter by the description of the mechanical/elastic behaviour of the OHAp/collagen matrix, many relevant phenomena acting at biochemical and biological level occurs at the interface between the mineral phase and the bioenvironment. In fact, viewing the bone tissue as a composite structure, its mechanical properties are dictated by those of the constituent components and of the organic/inorganic interface. For this reason, it is important to comprehend and model at atomic level how the protein matrix interacts with the biological apatite phase.

This branch of theoretical research is wide and covers both quantum mechanics and molecular mechanics methods applied on different surface models and (bio)molecules. In the present review, the most relevant results in this field will be presented, with a specific focus on those obtained at quantum mechanical level or theory. Indeed, the knowledge that can be obtained from first principle simulations (e.g., adsorbate/adsorbent internal geometries, adsorption energies, and so on) is mandatory to parametrize and develop force fields for inorganic/organic composites, which in turn could be confidently used in molecular mechanics modelling at the nano/mesoscale.

In general, when the goal of the simulation is describing the interaction between a mineral/material surface and a target molecule, it is important considering two questions, what is a physically sound starting molecule-to-substrate geometry and how many adsorbate molecules can be adsorbed by a given surface.

### 4.1. Interaction with Water

Let’s try answering the above concerns by considering a simple, yet very important molecule at the biological level, i.e., the water molecule. Indeed, since H_2_O is present in almost all biological fluids, this molecule controls most of the interactions between the components that build up life itself. In the case of mixed organic/inorganic systems such as bone tissues, where it is known that water may modify the mechanical properties of the composite and/or the interaction between the components, it is mandatory to understand the adsorption process of H_2_O onto the different OHAp surfaces. The considerations that will be presented in the following could be applied to more complex adsorbate as well.

For the first question, when designing a suitable starting geometry, it is commonly adopted the principle of complementarity between the electrostatic potential of the molecule and that of the adsorbant [151]. Both of them can be easily calculated from the analysis of the wave function of the two systems.

**Figure 7:**
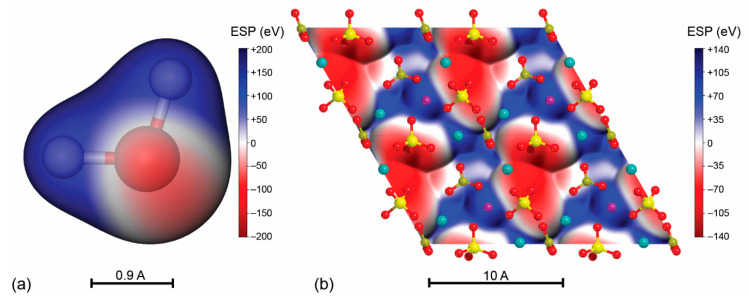
Electrostatic surface potential (ESP) of (**a**) water molecule and (**b**) of a (001) surface of type-AB carbonated hydroxylapatite, obtained at the DFT/B3LYP level of theory [153]. The three-dimensional maps were calculated on surfaces of constant electron density of 0.04 a.u. and 0.0001 a.u. for H_2_O and COHAp, respectively.

For example, water presents a negative region on the lone pairs of the oxygen atom and positive lobes on the hydrogen ones (Figure 7a). If the H_2_O molecule interacts with the (001) surface of type-AB carbonated hydroxylapatite (Figure 7b), one should expect that the negative regions of the surface will establish an interaction with the hydrogen atoms of the molecule, whereas the positive potential centred on the Ca^2+^ or other cations would be the preferred interaction site for the oxygen atom. Then, by optimizing the geometry of the adsorbate/adsorbant system, it is possible finding even more subtle interactions between the surface and the molecule, for example the formation of hydrogen bonds or the proton transfer from the molecule to the surface (chemisorption) [152].

For what regards the second question, the answer lies on the size of the adsorbed molecule(s) and the number of interaction sites available on the surface model, which in turn depends on the size of the model (single unit cell, supercells, etc.). For example, while several water molecules could be adsorbed on an area of about 9.307 × 9.307 Å^2^ (corresponding to that of a single unit cell) of hydroxylapatite, such surface extension would be too small to simulate the interaction with tropocollagen.

Several theoretical works (both at quantum mechanical and at molecular mechanics levels of theory) were devoted to the investigation of the surface features of different OHAp surfaces, particularly the (001), (010), (110), and (101) terminations, and their interaction with water [151,152,153,154,155,156,157,158,159,160,161]. The stability of the ‘dry’ stoichiometric surfaces of hydroxylapatite follows the order (001) > (101) > (110) > (010), an observation that dictates the crystal habit of the mineral. QM simulations carried out using the hybrid B3LYP functional revealed that H_2_O dissociates when it interacts with the (010) and (101) OHAp surfaces, which are the two most reactive ones [152,158,160]. Chiatti and co-workers [160] found and discussed different behaviours of the hydroxylapatite surfaces, in particular some surfaces, e.g., (001), decreased their reactivity towards water after the strongest interaction sites were covered, which is a typical behaviour of most of mineral surface. On the contrary, other surfaces, e.g., (010), showed an increased reactivity with increasing water loading, because, as suggested by the authors, the deformation of these surfaces due to the interaction with the solvent exposes other reactive ions to the environment. In general, the adsorption of water molecules is energetically favoured on the different OHAp surfaces, with energy values ranging from about −75 kJ mol^−1^ (high water coverage) to about −150 kJ mol^−1^ (low water coverage) [152,158,160]. By convention, negative adsorption energy values mean that the adsorbate is attracted by the surface.

**Figure 8:**
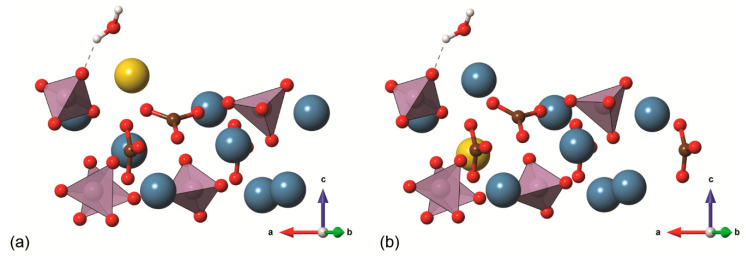
Adsorption of a single water molecule on the (001) surface of a mixed type-AB carbonated hydroxylapatite model. The difference between panels (**a**,**b**) resides in the position of the sodium ion (Na^+^/Ca^2+^ substitution), which is at the exposed at and below the mineral surface, respectively. Colour coding for atoms: dark cyan–Ca; yellow–Na; pink–P; red–O; ochre–C; white–H.

More recently, it was investigated by ab initio simulations the effect of type-A and/or type-B carbonate ion on the electrostatic surface potential (ESP) of (001) and (010) carbonated hydroxylapatite surface models and on the behaviour towards water adsorption [153,161,162]. Generally, and as expectable from a thermodynamic point of view, the energy required to cut a surface of carbonated hydroxylapatite is lower than the corresponding OHAp one because of entropy stabilization [161]. The type of carbonate ion substitution (A and/or B) exposed at the surface has a different modulation on the ESP, which is strong but confined in a small thickness of the mineral. As for stoichiometric hydroxylapatite, the (010) surface of COHAp has a higher reactivity than that of the (001) one, but the carbonate ions negligibly affect the surface relaxation [161]. Moreover, it was observed that a single molecule of water is strongly adsorbed by the (001) surface of both type-A type-AB carbonated hydroxylapatite [161,162]. For the sake of an example, Figure 8 graphically reports the adsorption configuration of H_2_O on the type-AB (001) COHAp surface. The interaction involved either Na^+^ or Ca^2+^, finding that water prefers with the latter as adsorption site. The results obtained in the different works suggested that the carbonate ion may lower both the adsorbate/surface interaction energy and the energy needed to deform the COHAp surface. It is also interesting that a significant difference in the adsorption energy of single H_2_O on (001) type-A carbonated apatite (−119 kJ mol^−1^) and (001) type-AB carbonated hydroxylapatite (−86 kJ mol^−1^) was observed, behaviour that can be ascribed to the stoichiometry of the simulated mineral surfaces. The quantum mechanical approach was definitely able to discriminate the adsorption properties of the two surfaces, which is an important predictive feature to design and develop biomaterials with tailored properties.

### 4.2. Interaction with Biomolecules

The importance in modelling and predicting the interaction between biological apatite and the bioenvironment becomes relevant when dealing with the simulations of the mineral/collagen/water system. This knowledge is nowadays pivotal in both biomaterial engineering and tissue mechanics, because it is relatively recent the recognition by Wang and co-workers of the effects of compositional changes in collagen content and collagen cross-linking in the risk of bone fracture [104]. For example, it was suggested that aging is accompanied by significant changes in the collagen properties, such as the decrease of mechanical strength, elastic modulus and toughness of the protein network up to 35%, 30% and 50%, respectively. Notwithstanding this important assessment, it is yet not fully clarified how the mechanical properties of collagen are depending by and controlled.

At the moment, most of the quantum mechanical characterizations of biomolecule/OHAp interactions fall in the gas-phase adsorption processes, i.e., the effect of water (solvent) is typically disregarded or heavily approximated, for example by employing continuum solvation models [163]. However, much useful information can be derived even from simplified models.

**Figure 9:**
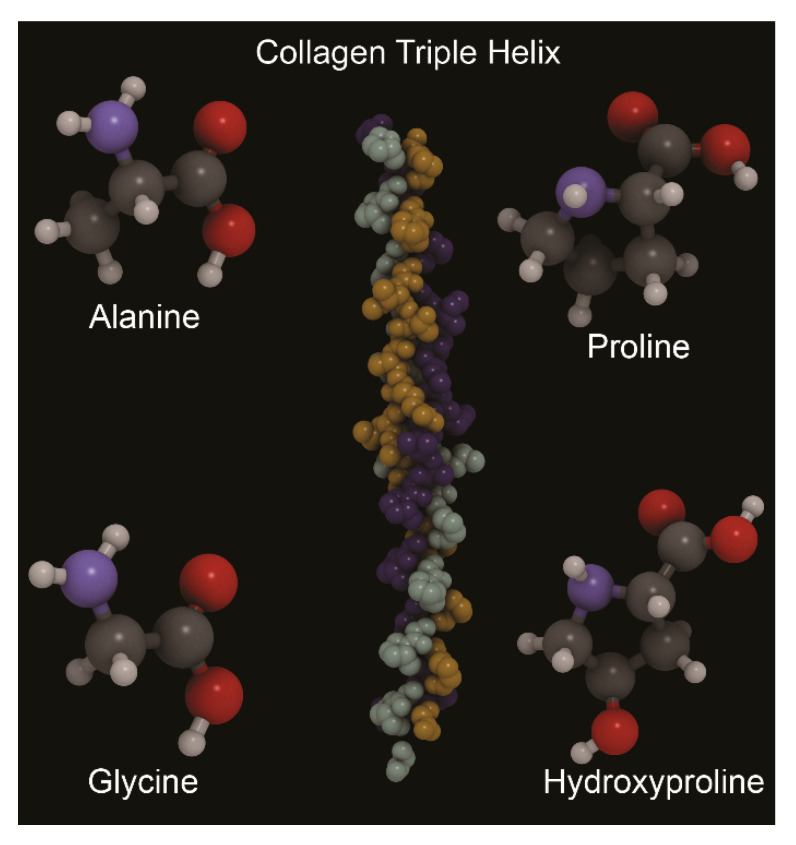
Structural model of a collagen triple helix, made from the Protein Data Bank code 1BKV [164], together with the four amino acids that form the sequence, i.e., glycine, alanine, proline and hydroxyproline. The protein was coloured to highlight the three winded chains. For the amino acids, carbon, nitrogen, oxygen and hydrogen were coloured in dark grey, blue, red and white, respectively.

As previously mentioned, first principle simulations of collagen are not feasible because of computationally too expensive. Just for the sake of an example, the collagen structure recorded in the PDB 1BKV [164] (see Figure 9) contains 563 atoms, excluding the hydrogen ones, which is a huge number from the QM perspective. However, it is possible to simulate the interaction between biological apatite models and both single amino acids and short peptide chains that are the basis of more complex collagen type-I triple helix (glycine, alanine, proline, and hydroxyproline).

The interaction between glycine and (001) and (010) hydroxylapatite surfaces were conducted independently by Rimola and co-workers [165] and Almora-Barrios et al. [166]. The former authors employed the CRYSTAL code and the B3LYP-D hybrid functional, whereas the second research group used the SIESTA code and the PBE functional. It is important to underline that, despite the difference in the DFT approaches, both authors obtained the same glycine/OHAp interaction geometry. In details, the molecule is more favourably adsorbed onto the (001) hydroxylapatite surface when in its zwitterionic state, establishing electrostatic COO^−^ ··· Ca^2+^ interactions and hydrogen bonds between the –NH_3_^+^ terminal and the oxygen atoms of the surface phosphate groups. Instead, the adsorption on the (010) OHAp face occurs with neutral glycine, which spontaneously transfers the carboxylic hydrogen atom to the surface, resulting in an electrostatic glycine^−^/OHAp^+^ pair. In both cases, very high adsorption energies were calculated, i.e., –306 kJ mol^−1^ and –381 kJ mol^−1^ for the glycine interactions with the (001) and (010) surfaces of hydroxylapatite, respectively.

In the above mentioned work, Almora-Barrios and co-workers [166] investigated also the adsorption of proline and hydroxyproline onto the same OHAp surfaces. The authors found that the adsorption of the two amino acids (and also glycine, see above) is energetically preferred occurring on the (010) surface, which is in agreement with the morphology of the mineral phase. In fact, as experimentally observed, bone mineral platelets are typically elongated along the c-axis, which means that, from the crystallographic point of view, they express more the (010) surface to the interaction with the bioenvironment and the (001) is less pronounced.

Corno and co-workers reported the simulation of lysine (Lys) and glutamic acid (Glu) on the same (001) and (010) surface of hydroxylapatite, within the DFT/B3LYP-D framework [158]. Since Lys is an amino acid with a basic character due to the –NH_2_ group on the side chain, it interacted with the lone pair of this nitrogen atom with the nearest calcium cation of both (001) and (010) OHAp surfaces. The behaviour of glutamic acid, which presents a carboxylic group in the side chain, was not so straightforward. In fact, while a proton transfer from the –COOH group of the side chain to a phosphate oxygen atom occurred for the Glu/(001) OHAp system, the same process was not observed for (010) surface of hydroxylapatite. As also stated by the authors, these results highlight the necessity to perform first principle simulations to carefully assess the behaviours and adsorption process involving different (bio)molecules and mineral surfaces.

Only about a decade ago, a first attempt to simulate the interaction between hydroxylapatite and a small chain of collagen, made of glycine–proline–alanine sequence, was performed at the DFT level by Aminova and collaborators [167]. Although the system was overly simplified, i.e., it was considered an intermolecular complex between the peptide fragment and a calcium ion, it provided information regarding the conformation of the biomolecule and the weak interactions between the small chain and the cation. The results showed the formation of a weak electrostatic bond between the Ca^2+^ ion and the oxygen atoms of the proline residue.

**Figure 10:**
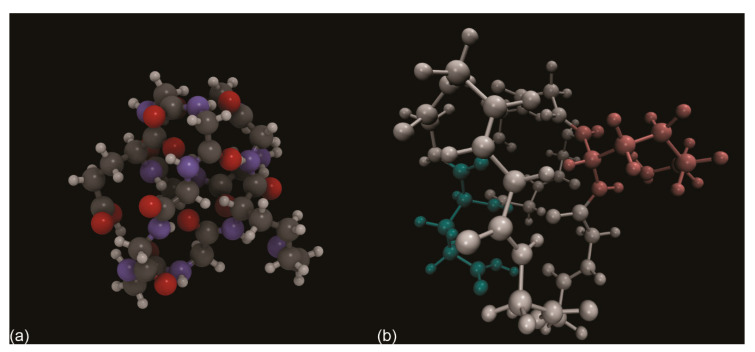
(**a**) Ball-and-stick models of the P1 protein structure optimized in the gas phase reported by Rimola and co-workers [168]. Carbon, nitrogen, oxygen and hydrogen were coloured in dark grey, blue, red and white, respectively. (**b**) Same P1 model as in (**a**), but with the amino acid residues coloured in white (Gly), red (Glu) and cyan (Lys) to better highlight their position in the folded structure.

Almost at the same time, a large-scale DFT/B3LYP-D simulation with the CRYSTAL code was performed on the possible peptide folding induced by the (001) and (010) surfaces of stoichiometric hydroxylapatite [168]. The protein chains employed in the cited work were relatively long (more than one hundred atoms), formed by a sequence of 12 glycine amino acids, which presented one (model P1) and two (model P2) residue mutations of glutamic acid and lysine. The amino acid sequence of peptide P1 was Gly-Gly-Lys-Gly-Gly-Gly-Gly-Gly-Gly-Glu-Gly-Gly, whereas for the P2 oligomer it was Gly-Gly-Lys-Gly-Gly-Lys-Glu-Gly-Gly-Glu-Gly-Gly. The topic covered in this work was understanding if the interaction between the biomolecules and the hydroxylapatite surfaces was able to overcome the energy cost necessary to unfold the small proteins. From the quantum mechanical analysis, it was found that in the gas phase the most stable conformation of both peptides is a folded, random coil structure, because it is stabilized by (i) the high number of intramolecular (H-bond) interactions and (ii) a more compacted conformation with respect to the helix that improve the effect of dispersive forces [168]. A graphical representation of the folded P1 Gly-Gly-Lys-Gly-Gly-Gly-Gly-Gly-Gly-Glu-Gly-Gly model is reported in Figure 10 for the sake of an example. Different hydration settings of the OHAp surfaces and/or the peptide were considered during the simulations, finding that the P1 oligomer is preferentially adsorbed in the random coil conformation. Instead, the P2 Gly-Gly-Lys-Gly-Gly-Lys-Glu-Gly-Gly-Glu-Gly-Gly peptide is more favourably adsorbed onto the hydroxylapatite surfaces in its helix conformation. For both random coil and helix conformation, the interaction with the apatite framework is given by electrostatic C = O ··· Ca^2+^ attraction and hydrogen bonding between amine (peptide) and phosphate (OHAp) groups, in agreement with previous observations with single amino acids. In addition, the proton transfer from the peptides to the hydroxylapatite surfaces was observed only when the biomolecules were in their helix conformation.

Albeit being very interesting and computationally intensive for a full quantum mechanical characterization of biomolecule/mineral surface interactions, this work did not consider a proper peptide chain resembling the type-I collagen found in the bone tissue.

**Figure 11:**
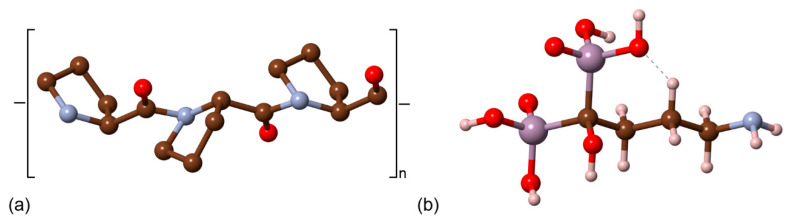
(**a**) PPII simplified collagen model employed by Cutini and co-workers [169], envisaging a monodimensional, polymer-like sequence of three proline amino acids. Hydrogen atoms were removed for the sake of clarity. (**b**) Alendronic acid model. Colour coding for atoms: carbon–ochre; nitrogen–light blue; phosphorous–pink; oxygen–red; hydrogen–white.

Very recently, it was proposed a simplified collagen model in interaction with the (010) hydroxylapatite surface [169]. The peptide was modelled as a type-II poly-L-proline polymer (labelled as PPII, see Figure 11a), whose conformation as a free system and adsorbed onto apatite was characterized by means of both static and dynamic simulations. In general, the PPII collagen model is favourably adsorbed on the hydroxylapatite surface with specific interactions similar to those discussed above. Among the many results reported by the authors, it was found that, during the adsorption process, (i) the polymer loses its helical form, with two residues parallel and one perpendicular to the surface, and (ii) the most exposed calcium cations of the OHAp surface are lifted because of the interaction with PPII.

Another important field of research related mineral-to-environment interactions is the investigation of the effects of (i) pharmaceutical drugs and (ii) adverse molecules on the properties of the biological apatite. For example, DFT simulations were conducted on the (001) surface of hydroxylapatite to characterize the adsorption of alendronic acid (AA, see Figure 11b), a well-known drug used to treat osteoporosis by inhibiting bone mineral resorption, and formic acid (FA), which simulates the (organic) acid environment in the mouth that may affect the enamel tissue of teeth [170]. In the first case, a very high adsorption energy in the AA/OHAp system (between −280 kJ mol^−1^ and −400 kJ mol^−1^) was observed, with the bisphosphonate molecule exhibiting a dissociative behaviour on the mineral surface (hydrogen atom transferred to either the apatite basic sites or the amine terminal of the drug molecule adsorbed on the neighbour cell). The surface protection activity of alendronic acid towards hydroxylapatite was suggested to reside in the possible multiple interactions of AA, namely with the mineral (electrostatic interaction) and between bisphosphonate molecules (hydrogen bonding).

The acid attack simulated via formic acid resulted in different adsorption mechanisms according to the involved OHAp crystal face [170]. On the (001) surface, a molecular adsorption was observed, with binding energy of about −190 kJ mol^−1^; conversely, a chemisorption process occurred on the (010) surface of hydroxylapatite (adsorption energy of ca. −250 kJ mol^−1^), with the FA molecule interacting as a formate ion. In general, the results showed that the acid molecule preferably binds to the Ca^2+^ cations, which represents important information concerning the processes that could trigger the dissolution of hydroxylapatite.

### 4.3. Interaction with Biomaterials

In the development of biomaterials, it is of utmost importance to increase their biocompatibility and osteoconductivity to both accelerate the bone/dental healing and reduce possible adverse effects (e.g., rejection of the implant). Among the different techniques to improve the cited properties of bone/dental implants is coating their surface with calcium phosphate materials (including OHAp), after appropriate pre-treatment to increase adhesion of the biomaterial with the implant. For the interested reader, this topic was addressed in a recent experimental review of Surmenev and co-workers [171]. Most implants are made of either titanium (Ti) or its alloys, because they present low density (hence, less stress shielding effect) and high strength, together with high corrosion resistance and non-toxicity. There are several ways to deposit OHAp on the surface of Ti-based implants, for example plasma spray, magnetron sputtering and electrophoretic deposition [171]. Hence, it is important characterizing not only the interaction between hydroxylapatite and the biological environment, but also its interface with metallic and other biomaterials.

The study of metal/OHAp composite is relatively recent from the computational point of view because of the high computational resources required by the simulations, and just few works are present in literature. Interfacing a mineral/ceramic phase with hexagonal lattice (OHAp) with a metal/alloy typically characterized by a cubic lattice is not straightforward. The accommodation of the two materials in contact with each other without too much tensile/compressive strains in one or both of them may require large cells with hundreds or thousands of atoms. One of the first works in this sense was proposed by Allenstein and co-workers, who performed a combined experimental/theoretical study of the structural and adhesion properties of hydroxylapatite coating on two ferromagnetic shape-memory alloys, Ni−Mn−Ga and Fe−Pd. The authors considered experimental delamination tests on sputter-deposited thin films of OHAp, and performed DFT simulations on simplified models of OHAp/alloy interfaces using the PWSCF code, PBE functional, and plane-wave basis sets [172]. The authors measured for the OHAp/Ni–Mn–Ga system a delamination work of 1.6 J m^−2^, with failure occurring at the composite interface, and a theoretical adhesion of −1.9 J m^−2^. Conversely, the delamination test performed on the OHAp/Fe–Pd composite showed the rupture within the hydroxylapatite coating, meaning that the interface is mechanical stronger than stoichiometric OHAp. The DFT simulations provided data in agreement with the experimental findings, with a calculated rupture force of about 1.8 GPa.

More recently, Grubova and collaborators performed ab initio DFT calculations of the interaction between rutile (the TiO_2_ phase stable in ambient conditions) and either stoichiometric [173] or Si-doped hydroxylapatite [174], to obtain an atomistic insight of implant protection from failure. Both studies were carried out using the VASP code, projector-augmented wave basis sets, and the PBE functional, and exploited a “melt and quench” approach. Briefly, this method involves several heating and cooling cycles of the materials, performed with molecular dynamics protocols. The authors employed a ReaxFF (reactive force field) parametrization for both rutile and stoichiometric/Si-doped hydroxylapatite. In both works [173,174], the work of adhesion, structural and charge density analyses were performed, observing that there are significant distortions surficial atoms of rutile and that, at the interface, there is the formation of Ca–O and Ti–O bonds. Furthermore, the replacement of some PO_4_ groups with SiO_4_ ones at the apatite/rutile interface has a large impact on its adhesion and mechanical properties.

## 5. Conclusions and Future Perspectives

The present review was intended to highlight the results obtained with computational simulations at the atomistic level, from few to thousands of atoms in the modelled systems, carried out on the bone and dental mineral phase, namely hydroxylapatite and biological apatite. Two of the main adopted methodologies, i.e., first principle quantum-mechanical approaches and molecular mechanics technique, were presented, showing their merits and limitations in this research field.

The reported studies are many, as are the topics covered by them, from the crystal-chemical and surface properties of the mineral phase, to the mechanical failure of the OHAp/collagen composite. The employed approaches proved to be solid in providing results that can be well correlated with experimental ones, investigating phenomena occurring at different time and length scales and providing further atomistic details that could explain the architecture and the behaviour of hard tissues.

Considering future perspectives of the present work, is the in-silico approach here presented able to tackle the complexity of biological systems? Is it fast enough? Looking at the results obtained in the last decade, it is possible to assess that the simulation methods, corroborated with past and future experimental findings, are suitable for improving our understanding on this matter. It is worth remembering that large systems are usually accompanied by extremely high computational costs if the objective is obtaining quantum-mechanical (very accurate) data, but (i) the simulations could be focussed on specific, smaller sites of the overall model and (ii) more approximation could be employed. Each solution has its drawback, of course: in case (i), the overall picture and long-range effects could be lost, whereas in (ii) the penalty resides in the accuracy of the results.

However, the authors are confident that in future it could be possible to overcome some of these issues in a reliable way. Indeed, the computing architectures are experiencing fast developments, and the introduction of accelerators (graphical processor unit computing and many-core processors, just to cite a couple of well-known examples) has further improved the speedup of the simulations and augmented the size of the systems that can be studied. At the same time, mathematical algorithms, numerical libraries and codes for quantum mechanical and molecular mechanics are under constant development to fully exploit the available computing architecture. In fact, while most of the basic theoretical framework behind QM and MM simulations was already known more than half a century ago, the codes are always evolving. On one side, they undergo technical adaptation for the emerging computing hardware, whereas on the other side more and more features (e.g., DFT functionals, facilities to model complex systems, and so on) are included to provide more flexibility for the computational parameters during the simulations.

In addition, the mathematical algorithms forming the backbone of machine learning (a branch of artificial intelligence) and the associated technology are growing at a fast pace since about five years. These methods have been applied to DFT for high-throughput simulations for relatively simple systems (e.g., small cubic minerals), providing huge amount of data [175,176,177]. It is highly probable that, in the future, it will be possible to extend machine learning approaches to such complex systems as hard tissues, finding new insights and correlations that could be of extreme help for several fundamental and applied fields, such as biology, medicine, and materials science.

## Figures and Tables

**Table 1:** Lattice parameters and bond lengths of hydroxylapatite (OHAp), type-A carbonated apatite (CAp) and mixed type-AB Na-bearing carbonated (hydroxyl)apatite [Na-C(OH)Ap] models, compared to similar structures experimentally determined by X-ray diffraction (XRD) and neutron diffraction.

	OHAp				Type-A CAp			Type-AB Na-C(OH)Ap			
	B3LYP	B3LYP-D*	XRD	Neutron	B3LYP	B3LYP-D*	XRD	B3LYP ^a^	B3LYP-D*	B3LYP ^b^	XRD
Reference	[61]	[68]	[69]	[70]	[61]	[71]	[66]	[62]	[72]	[62]	[63]
S.g.	*P6_3_*	*P6_3_*	*P6_3_/m*	*P6_3_/m*	*P1*	*P1*	$P\bar{3}$	*P1*	*P1*	*P1*	$P\bar{3}$
*a* (Å)	9.433	9.385	9.417	9.425	9.582	9.584	9.521	9.395	9.336	9.451	9.386
*b* (Å)	9.433	9.385	9.417	9.425	9.764	9.631	9.521	9.337	9.293	9.482	9.386
*c* (Å)	6.896	6.871	6.875	6.884	6.877	6.859	6.873	6.904	6.875	6.897	6.914
α (°)	90.0	90.0	90.0	90.0	89.3	89.6	90.0	89.6	89.7	89.5	90.0
β (°)	90.0	90.0	90.0	90.0	89.8	89.2	90.0	90.4	90.5	90.1	90.0
γ (°)	120.0	120.0	120.0	120.0	121.9	121.8	120.0	119.8	119.8	120.6	120.0
V (Å^3^)	532	524	528	530	546	538	540	526	518	532	528
P–O (Å)	1.551	1.550	1.532	1.536	1.540	1.553	1.53	1.552	1.550	1.548	1.513
O–H (Å)	0.97	0.97	1.09	0.90	−	−	−	−	−	0.97	−
Ca–O (Å)	2.391	2.464	2.398	2.400	2.390	2.341	2.34	2.422	2.439	2.415	2.360
C–O (Å)	−	−	−	−	1.290	1.285	1.24	1.296	1.294	1.291	−

^a^–results related to a single crystallographic cell; ^b^–results calculated with a 2 × 2 × 1 supercell.

**Table 2:** Lattice parameters and selected ion content (in wt.%) in different types of calcium apatite models, compared to experimental results reported in literature.

	Theoretical Models					Experimental ^c^		
	OHAp ^a^	Type A1	Type B	Type A–B	Type A–B	Bone	Dentin	Enamel
		Cap ^a^	Na-COHAp ^b^	Na-Cap ^b^	Na-COHAp ^b^			
*a* (Å)	9.433	9.582	9.3982	9.4027	9.4493	9.41	9.421	9.441
*b* (Å)	9.433	9.764	9.3327	9.3707	9.4486	9.41	9.421	9.441
*c* (Å)	6.896	6.877	6.9071	6.9134	6.9028	6.89	6.887	6.88
α (°)	90	89.3	90.3	89.3	89.6	90	90	90
β (°)	90	89.8	90	90.7	90.2	90	90	90
γ (°)	120	121.9	120.6	120	120.3	120	120	120
V (Å^3^)	531.5	546	521.3	527.4	532.2	528.4	529.4	531.1
Ca^2+^	39.89	38.81	37.87	36.79	39.07	34.8	35.1	36.5
P	18.05	18.05	16.26	15.84	17.86	15.2	16.9	17.7
CO_3_^2-^	−	5.82	6.3	12.26	3.01	7.4	5.6	3.5
Na^+^	−	−	2.41	2.35	0.58	0.9	0.6	0.5

^a^–Ref. [61]; ^b^–Ref. [62]; ^c^–Refs. [6,8,73].

**Table 3:** IR and Raman vibrational modes (in cm^−1^) of the phosphate group of hexagonal hydroxylapatite (space group *P6*_3_) calculated at the DFT level of theory, compared with experimental results.

Normal Mode	IRREP	IR Active	Raman Active	Theoretical		Experimental	
				B3LYP-D* [90]	B3LYP [89]	IR [74]	Raman [91]
ν_2_ (PO_4_)	*A*	✓	✓	465	459		432
	*A*	✓	✓	494	489		454
	*E*_1_	✓	✓	441	437		
	*E*_1_	✓	✓	478	475		432
	*E*_2_	x	✓	442	440	462	449
	*E*_2_	x	✓	460	457		
ν_4_ (PO_4_)	*A*	✓	✓	568	566	566	
	*A*	✓	✓	600	599	660–520	593
	*A*	✓	✓	623	621		609
	*E*_1_	✓	✓	575	573		
	*E*_1_	✓	✓	601	600	632	
	*E*_1_	✓	✓	611	609	602	
	*E*_2_	x	✓	567	566		581
	*E*_2_	x	✓	590	588		609
	*E*_2_	x	✓	635	633		617
ν_1_ (PO_4_)	*A*	✓	✓	975	972		962
	*E*_1_	✓	✓	976	972	962	
	*E*_2_	x	✓	978	974		962
ν_3_ (PO_4_)	*A*	✓	✓	1057	1054	1190–976	1034
	*A*	✓	✓	1069	1064		1048
	*A*	✓	✓	1102	1096		1077
	*E*_1_	✓	✓	1050	1045		1043
	*E*_1_	✓	✓	1072	1068	1042	
	*E*_1_	✓	✓	1114	1110	1091	
	*E*_2_	x	✓	1046	1041		1028
	*E*_2_	x	✓	1081	1077		1055
	*E*_2_	x	✓	1086	1081		1077

**Table 4:** IR and Raman vibrational modes (in cm^−1^) of the carbonate ion in different models of carbonated (hydroxyl)apatite, C(OH)Ap, as calculated from DFT simulations at the B3LYP level of theory. Results from experimental techniques are reported for the sake of a comparison.

Mode		DFT					Experimental		
	Type-A Cap ^a^	Type-A Cap ^b^	Type-B COHAp ^b^	Type-B COHAp ^c^	Type-AB Cap ^b^	Type-AB COHAp ^b^	FTIR ^d^	μ-FTIR ^e^	Raman ^f^
ν_4a_ (CO_3_)–A	677	−	−	−	660	666	−	670	676
ν_4b_ (CO_3_)–A	784	−	−	−	759	784	−	750	754
ν_2_ (CO_3_)–A	870	826	−	−	890	889	880	878	−
ν_1_ (CO_3_)–A	1135	−	−	−	1086	1128	−	−	1103
ν_3a_ (CO_3_)–A	1524	1422	−	−	1442	1503	1457	−	−
ν_3b_ (CO_3_)–A	1617	1512	−	−	1589	1612	1550	−	−
ν_4a_ (CO_3_)–B	−	−	−	706	693	693	−	670	689
ν_4b_ (CO_3_)–B	−	−	−	715	721	719	−	750	718
ν_2_ (CO_3_)–B	−	−	813	876	875	879	876	871	−
ν_1_ (CO_3_)–B	−	−	−	1109	1091	1099	−	−	1073
ν_3a_ (CO_3_)–B	−	−	1376	1481	1463	1472	1418	−	−
ν_3b_ (CO_3_) -B	−	−	1416	1562	1545	1568	1462	−	−

^a^–Ref. [89]; ^b^–Ref. [94]; ^c^–Ref. [62]; ^d^–Ref. [98]; ^e^–Ref. [100]; ^f^–Ref. [99].

**Table 5:** Second-order elastic moduli of stoichiometric hydroxylapatite theoretically calculated at DFT level with the PBE functional, using Gaussian-type orbitals (GTO) and planewave (PW) basis sets. Experimental results are reported for a comparison.

Moduli (GPa)	GTO–AE		GTO–ECP		PW				Experimental	
	PBE ^a^	PBE-D ^a^	PBE ^a^	PBE-D ^a^	PBE ^a^	PBE-D ^a^	PBE ^b^	PBE ^c^	^d^	^e^
*C*_11_	171.5	182.1	226.0	236.5	126.9	147.8	118.3	117.7	143.4	137
*C*_12_	63.6	70.1	81.6	86.4	37.6	49.7	31.6	31.1	44.5	42.5
*C*_13_	76.0	80.0	83.7	86.0	67.1	64.1	63.7	66.4	57.5	54.9
*C*_33_	207.0	222.2	262.2	278.0	170.0	186.2	156.8	165.0	180.5	172
*C*_44_	28.9	36.1	43.3	50.2	41.8	45.6	33.5	38.5	41.5	39.6
*C*_66_	53.9	56.0	72.2	75.1	44.3	48.9	*43.4*	*43.3*	49.5	47.3
*RMSE*	19.9	27.3	51.8	59.8	9.6	4.8	15.8	14.2	−	−

^a^–Ref. [68]; ^b^–Ref. [107]; ^c^–Ref. [110]; ^d^–Ref. [108]; ^e^–Ref. [109]. AE and ECP stand for all-electron and effective-core pseudopotentials basis sets for the description of the core electrons of calcium atoms. PBE-D refers to the DFT functional corrected for dispersive forces with the Grimme’s approach [115]. Root mean square errors (RMSEs) with respect to the experimental results of Ref. [109] are also reported.

## Data Availability

The data are available within the article.
